# α-linolenic acid-induced facilitation of GABAergic synaptic transmission is mediated via acid-sensing ion channel (ASIC1a) activity in the basolateral amygdala

**DOI:** 10.3389/ebm.2025.10545

**Published:** 2025-05-15

**Authors:** Volodymyr I. Pidoplichko, Taiza H. Figueiredo, Maria F. M. Braga, Ann M. Marini

**Affiliations:** ^1^ Department of Anatomy, Physiology and Genetics, Uniformed Services University of the Health Sciences, Bethesda, MD, United States; ^2^ Department of Neurology, Neuroscience, Molecular and Cellular Biology, Graduate School of Nursing, Uniformed Services University of the Health Sciences, Bethesda, MD, United States

**Keywords:** alpha-linolenic acid, rat, GABA, inhibitory bursts, ASIC1a channels

## Abstract

Epilepsy affects more than 70 million people worldwide. A seizure focus that develops in different cortical brain regions can present as either focal or generalized seizures. Temporal lobe epilepsy is a highly pharmacoresistant form of epilepsy that involves the amygdala, hippocampus with or without hippocampal sclerosis as well as other limbic structures. Loss and/or dysfunction of GABAergic inhibitory neurons play a critical role in tipping the balance toward excitation. Synchronous burst firing is a feature of inhibitory neurons that is thought to regulate and rectify large excitatory neuronal networks in the BLA and is thought to underlie higher cognitive function. Acid sensing ion channels (ASIC) activated by decreases in pH, the presence of ammonium ion or a slight lowering of temperature are present on excitatory and inhibitory neurons and can alter excitability. The net effect of the activation of ASIC1a channels in the BLA is inhibition. ASIC1a channels are active in the basal state, enhancing primarily GABAergic inhibition by direct depolarization of interneurons but also by indirect excitation of interneurons via ASIC1a-mediated depolarization of pyramidal neurons. In this study, we examine the contribution of ASIC1a channel activation on alpha-linolenic acid (ALA)-induced GABAergic inhibitory synchronous burst firing in the BLA. Our results show that ALA initiates inhibitory bursts that are dependent, in part, on the activation of ASIC1a channels that may in turn be mediated by mature brain-derived neurotrophic factor.

## Impact statement

Hyperexcitability is associated with informational processing deficits that may lead to disconnection and clinical cognitive impairment. Brain injuries caused by acute and chronic neurological disorders can impair neuronal function and/or lead to neuronal loss. GABAergic inhibitory neurons carry out diverse functions in brain. One major function of GABAergic inhibitory interneurons is to arrange and generate oscillations. Oscillations in the brain can either synchronize or de-synchronize neural ensembles. Loss or dysfunction of GABAergic inhibitory neurons may contribute to epilepsy and lead to the disruption of oscillations in the BLA. The novel finding that alpha-linolenic acid facilitates inhibitory bursts suggests that this nutraceutical may compensate for the loss and/or dysfunction of inhibitory neurons to reduce seizures and restore oscillatory function.

## Introduction

Epilepsy is one of the most common neurological disorders and affects more than 70 million people around the world. Epilepsy, defined as spontaneous recurrent seizures or as two unprovoked seizures separated by more than twenty-four hours, is commonly treated with anticonvulsant therapy. There are approximately 150,000 individuals that have experienced one unprovoked seizure in the United States [1] and those that have had a brain insult, an electroencephalogram (EEG) with epileptic discharges or an abnormality on brain imaging and a nocturnal seizure are at greatest risk of having a second unprovoked seizure over the next 2 years [2]. Whether to treat a single seizure requires clinical judgement in weighing the risks of having a second seizure versus anticonvulsant side effects [2]. There are social implications for anyone that has had their first seizure including loss of driving privileges and potential employment issues.

Epilepsy has intriguing features beginning with epileptogenesis, the process of converting a normal functioning brain with a variable latent period without seizures into one that generates spontaneous recurrent seizures. A seizure is a transient synchronous discharge of neuronal activity in the brain but the process of conversion to an epileptic state is complicated. In fact, the details of how normal neuronal circuits develop into transient abnormal synchronous discharges are unknown. The pathophysiology has been attributed, at least in part, to the imbalance of excitatory and inhibitory neurons and/or function in the brain. However, this is not the entire story. Absence seizures, for example, result from an aberrant increase in inhibition due to impaired uptake of γ-aminobutyric acid [GABA] [3] although recent evidence suggests that a reduction in cortical inhibition may be a significant contributing factor in these generalized seizures [4].

The amygdala is an almond-shaped structure that is located in the mesial temporal lobe of the brain [5]. At least thirteen different nuclei define the amygdala where they carry out diverse functions including emotional memory, and normal behavioral functions [6]. The basolateral division of the amygdala (BLA) is a relatively new division of the amygdala that is associated with the cortex [6]. The BLA has reciprocal connections with the ventral hippocampus and prefrontal cortex, areas critically involved in fear memory, among others [7–9].

The amygdala plays a fundamental role in temporal lobe epilepsy [10, 11]. Temporal lobe epilepsy (TLE) is the most common type of focal epilepsy and, in the presence of hippocampal sclerosis, at least one-third of patients suffer from pharmacoresistance [12, 13]. The amygdala is one of the brain regions that shows extensive damage in patients with TLE [11, 14–16]. Studies show that the seizure focus resides in the amygdala and/or hippocampus although the seizure focus is most commonly found in both brain regions [11, 17, 18].

The amygdala, however, may be the most common brain region of the seizure focus. It is well-known that kindling, the use of repeated electrical stimulation in laboratory animals, results in spontaneous recurrent seizures much faster when performed in the amygdala compared with the hippocampus [19, 20]. Moreover, the earliest indication of interictal spike activity or epileptiform discharges occurs in the amygdala and piriform cortex even if kindling was performed in the hippocampus [21, 22]. Seizure spread from the amygdala to other areas may be due to its extensive reciprocal connections to temporal and other cortical brain regions [23] that facilitates the spread of seizures to the hippocampus and to other brain regions. The mechanisms of the vulnerability of the amygdala to the generation of seizures are largely unknown [11, 24].

The BLA plays an important role in the normal and abnormal functions of the amygdala. Sensory information from thalamocortical areas project to the BLA [25, 26]. Importantly, BLA activation is particularly responsible for the generation of status epilepticus in animal models of seizures even when the seizures are generated in extra-amygdalar brain regions [27, 28]. In addition, prolonged electrical stimulation of the amygdala sets off status epilepticus more readily when it is done in the BLA compared with other areas of the amygdala [29]. However, the reasons behind the susceptibility of the BLA in the generation of status epilepticus are unclear.

The BLA contains two types of neurons, glutamatergic pyramidal (principal) neurons and γ-amino-butyric acidergic (GABAergic) inhibitory neurons [30, 31]. The vast majority of neurons in the BLA are glutamatergic or principal neurons (80–85%) whereas GABAergic inhibitory neurons represent 15–20% of neurons [5, 32, 33]. GABAergic inhibitory interneurons co-express neuropeptides such as the calcium binding proteins calbindin or calretinin, cholecystokinin (CCK), vasoactive intestinal peptide (VIP), somatostatin or parvalbumin [34–38]. Interestingly, GABAergic inhibitory neurons co-expressing the neuropeptide parvalbumin comprise about 40% of the total number of GABAergic inhibitory neurons and are the principle foundation of perisomatic innervation of principal neurons that may be involved in feedback inhibition in the BLA. In contrast, GABAergic inhibitory interneurons that co-express calretinin make up about 25–30% and mostly synapse on other inhibitory interneurons [37, 39–41].

GABAergic inhibitory interneurons tightly regulate the excitability of the BLA [42], despite representing only about 20% of the total number of neurons. The GABA_A_ receptor mediates fast inhibitory synaptic neurotransmission but there are modulators that also regulate neuronal excitability in the BLA. The glutamate receptor subtype, kainic acid, is involved in synaptic transmission and modulates the presynaptic release of glutamate [43] and GABA [44–47]. Moreover, kainic acid is involved in epilepsy [24, 48, 49]. The kainic acid receptor consist of five different subtypes: Gluk1-3 (previously called GluR5-7), and GluK4-5 (previously called KA1-2) [50]. Kainic acid receptors are tetramers forming homomeric or heteromeric receptors; GluK4-5 subunits form functional receptors only in combination with GluK1-3 subunits [51, 52]. The N-terminal amino terminal domain (ATD) plays a major role in the assembly of heterodimers and homodimers because the formation of dimers begins at the ATD domains [53]. Alternative splicing and mRNA editing alter substrate binding and ion fluxes [51, 54, 55]. Thus, the combination of subunits results in a diverse complement of distinct receptors with different pharmacological and biophysical properties. High levels of mRNA coding for GluK1-3 are expressed in the amygdala [56–58]. The mRNA levels of GluK1 are especially elevated in the BLA, higher than in the hippocampus [56, 57]. It has been shown that GluK1-containing kainate receptors contribute to excitatory postsynaptic currents (EPSCs) when recorded from BLA glutamatergic neurons [59, 60] and increases the amplitude and frequency of action-potential spontaneous GABAergic inhibitory postsynaptic currents (IPSCs) recorded from BLA excitatory neurons [57].

Several studies implicate the GluK1-containing kainate receptors in temporal lobe epilepsy or complex partial seizures. ATPA, a GluK1 agonist, induces spontaneous epileptiform bursting in rat amygdala slices [56], and limbic status epilepticus when administered intravenously or when the compound is directly injected into the rat amygdala [24, 49]; the seizure-generating effects of ATPA are blocked by the GluK1 antagonist, LY293558 [61]. Also, antagonists of GluK1-containing receptors block limbic seizures beginning in the hippocampus induced by pilocarpine, a muscarinic agonist, or electrical stimulation *in vitro* or *in vivo* [24, 48]. Topiramate, a GluK1 antagonist [60], prevents ATPA-induced seizures but has no anti-seizure effect on other ionotropic glutamate receptor subtypes [49] i.e., NMDA or α-amino-3-hydroxyl-5-methyl-4-isoxazole-propionate (AMPA). These results suggest that blocking GluK1-containing kainic acid receptors is a major mechanism of stopping seizures by topiramate. Expression levels of GluK1 are markedly increased in epileptic temporal lobe brain regions in human as well as in rats [62, 63]. Although GluK1-containing receptors can increase EPSCs, and therefore, excitability, in glutamatergic neurons and increase the release of GABA from presynaptic terminals of GABAergic inhibitory neurons to reduce excitability of glutamatergic neurons at low glutamate concentrations, elevated concentrations of glutamate as occurs during a seizure suppresses the release of GABA from presynaptic terminals [57] thereby exacerbating seizure activity. Additional studies have shown that the overall effect of elevated activation of GluK1-containing kainate receptors is a striking increase in neuronal excitability in the BLA and generation of spontaneous epileptiform discharges [61].

Physiological synchronous burst firing, a property of inhibitory neurons [64–66], resets and controls excitatory activity [64, 67]. As a result, GABAergic inhibitory interneurons play a central role in arranging and generating oscillations [68–71]. Synchronous oscillations in the BLA appear to be important for safety perception [72] and fear response [73]. Recently, spontaneous rhythmic oscillatory GABA_A_ receptor-mediated inhibitory bursts were recorded with an average burst frequency of 0.5 Hz from the rat BLA that were dependent upon NMDA receptor activation, specifically the NR2A subunit, located on GABAergic inhibitory neurons [74]. However, the role of other receptors and/or channels in the generation of inhibitory burst activity is unknown.

A small reduction in the pH or temperature, or the presence of ammonium ion activates H+-gated sodium channels called acid-sensing ion channels (ASICs) [75, 76]. The channel was cloned from rat brain and three types of ASIC channels have been identified, ASIC1, ASIC2 and ASIC [3, 77]. ASICs are members of the epilthelial/degenerin sodium channel family with different biophysical properties. ASIC1 has two splice variants, ASIC1a and ASIC1b; ASIC1a is broadly distributed in brain with the highest expression found in the amygdala, among other brain regions [76, 78]. Pidoplichko et al., (2014) [79] demonstrated that activation of ASIC1a channels by a reduction in pH or in the presence of ammonium evokes inward currents depolarizing excitatory neurons and interneurons and enhanced IPSCs more than EPSCs from excitatory neurons and increase inhibitory activity in the BLA by the activation of inhibitory neurons and indirect activity by the synaptic activation of glutamatergic neurons. Pharmacological manipulation in rat BLA slices to induce epileptiform activity using elevated potassium or low magnesium, a strategy that relieves the magnesium block of NMDA receptors to increase activity, was completely blocked by ammonium [79]. These results confirm that activation of ASIC1a channels enhance inhibition over excitation in the BLA.

We have shown previously that alpha-linolenic acid (ALA), an omega-3 polyunsaturated fatty acid (PUFA), increases the facilitation of GABA_A_ receptor-mediated neurotransmission in the BLA [80]. An increase in GABA_A_ receptor-mediated neurotransmission induced by mature brain-derived neurotrophic factor (mBDNF) elicited a similar increase in inhibitory activity. These results suggest that ALA may protect neurons via a bidirectional effect by reducing excitation through activation of a background potassium channel [81] and enhancing inhibition [80]. We now show that ALA enhances inhibitory burst activity in the BLA by activating ASIC1a channels possibly via a mBDNF-mediated mechanism.

### Ethics statement

The experiments followed the Guide for the Care and Use of Laboratory Animals (Institute of Laboratory Animal Resources, National Research Council) and were in accordance with the guidelines and approved by the Uniformed Services University of the Health Sciences Institutional Animal Care and Use Committees (IACUC). The animal care and use programs of both institutions are accredited by the Association for Assessment and Accreditation of Laboratory Animal Care International.

### Animals

Experiments were performed using 8–16 weeks old male, Sprague–Dawley rats (Charles River, Wilmington, MA, United States). Rats were pair-housed on arrival and acclimated for 3 days. A total of ten rats were used for the study. Animals were housed on an environmentally controlled room (20–23°C, ∼44% humidity, 12-h light/12-h dark cycle [350–400 lux], lights on at 6:00 am), with food (Harlan Teklad Global Diet 2018, 18% protein rodent diet; Harlan Laboratories; Indianapolis, IN) and water available *ad libitum*. All rats used were not injected with substances prior to electrophysiological experiments.

### Electrophysiological experiments

The procedures for obtaining the whole-cell recordings from the BLA region have been previously described [80, 82]. The rats were anesthetized with isoflurane before decapitation. Coronal brain slices (400 µm thick) containing the amygdala were cut in ice-cold solution (in mM: 115 sucrose; 70 N-methyl-D-glucamine-gluconate (NMDG); 1 KCl; 2 CaCl_2_; 4 MgCl_2_; 1.25 NaH_2_PO_4_; 30 NaHCO_3_) with the use of a vibratome (Leica VT 1200 S; Leica Microsystems, Buffalo Grove, IL, United States). The slices were transferred to a holding chamber at room temperature of about 23°C, in a bath solution containing (in mM): 125 NaCl; 2.5 KCl; 1.25 NaH_2_PO_4_; 21 NaHCO_3_; 2 CaCl_2_; 1 MgCl_2_; and 25 D-glucose. The recording solution (artificial cerebrospinal fluid; ACSF) was the same as the holding bath solution. All of the solutions were saturated with 95% O_2_/5% CO_2_ to achieve a pH near 7.4. The recording chamber (0.7 mL capacity) had continuously flowing ACSF (∼8 mL/min) at 30–31°C. The osmolality of the ACSF was adjusted to 325 mOsm with D-glucose.

To visualize the neurons, we used a ×40 water immersion objective equipped with a CCD-100 camera (Dage-MTI, Michigan City, IN, United States), under infrared light, using Nomarski optics of an upright microscope (Zeiss Axioskop 2, Thornwood, NY, United States).

The recording electrodes had resistances of 3.5∼4.5 mW when filled with the internal solution (in mM): 60 CsCH_3_SO_3_; 60 KCH_3_SO_3_; 5 KCl; 10 EGTA; 10 HEPES; 5 Mg-ATP; 0.3 Na_3_GTP (pH 7.2; osmolality was adjusted to 295 mOsm with potassium gluconate). Tight-seal (over 1 Giga Ohm) whole-cell recordings were obtained from the cell body of the principal neurons, distinguished from the interneurons by their larger size, pyramidal shape, and electrophysiological characteristics. Access resistance not exceeding 20 Mega Ohms was monitored during the recordings, and the cells were rejected if the resistance changed by more than 15% during the experiment. The currents were amplified and filtered (2 kHz) using the Axopatch 200B amplifier (Axon Instruments, Foster City, CA, United States) with a four-pole, low-pass Bessel filter, digitally sampled (up to 2 kHz) using the Clampex 10.7 software (Molecular Devices, Sunnyvale, CA, United States), and subsequently analyzed using Origin2019b software (OriginLab Corporation, Northampton, MA, United States).

GABA_A_ receptors (GABA_A_Rs)-mediated sIPSCs were recorded in a voltage-clamp mode at holding potential (Vh) of + 30 mV in the presence of D-AP5 (50 μM); SCH50911 (10 μM); LY341495 (3 μM). After a BLA cell was patch-clamped, the holding potential was switched from conventional − 58 to + 30 mV. The cell was left to equilibrate with the new Vh for about 4 min in drug-free bath solution (ACSF) and then another 4 min in antagonists-containing bath solution. Spontaneous IPSCs were recorded after that. Pressure-application of substances was performed with the help of the technique described previously [83]. Substances used in this study were as follows: D-AP5, a competitive NMDA receptor antagonist, NMDA, Ammonium chloride and all chemicals used for buffers were purchased from Sigma-Aldrich Chemical Co (St. Louis, MO). Mature BDNF and Ibuprofen were purchased from Tocris Bioscience, (Ellisville, MO). Alpha-linolenic acid (ALA) was purchased from Nu-Chek Prep Inc (Elysian, MN) and was freshly prepared on the day of experimentation. ALA was dissolved in ethanol at a molar concentration and then diluted in ACSF solution to reach a final concentration of 50, 100 or 200 μM. It was reported that ALA undergo auto-oxidation [84], therefore all manipulations of ALA were made under nitrogen. The experiments were performed in the presence of the NMDA receptor antagonist D-AP5, unless specified in the description of results and figure legend.

### Statistical analysis

Statistical values are presented as means ± standard error (SE) of the mean, and comparisons were made using paired-t test. Results were considered statistically significant when the p value was <0.05. Sample sizes (n) refers to the number of currents.

## Results

**FIGURE 1:**
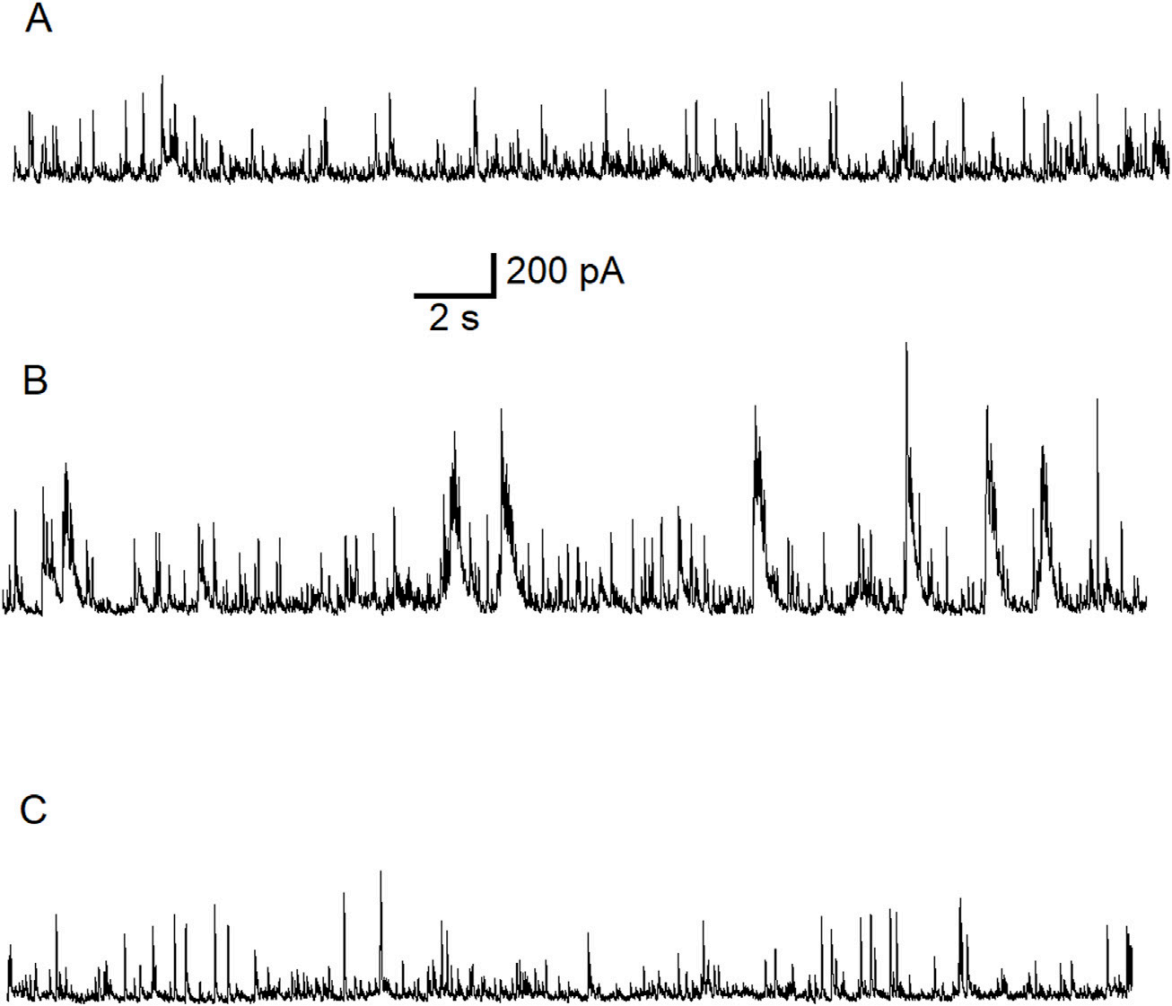
Typical potentiating effect of bath-applied ALA on inhibitory activity mediated via GABA_A_Rs. The experiment was conducted at Vh = +30 mV in the presence of NMDARs antagonist D-AP5 (50 µM). Control current traces are demonstrated **(A)**. Bath-applied ALA (50 µM) facilitated inhibitory activity (bursts) **(B)**. Note the initiation of inhibitory bursts in the presence of the NMDA receptor antagonist D-AP5. Inhibitory activity subsided during wash-out of ALA **(C)**.

In the first experiment we replicate the effects of ALA on GABAergic neurotransmission in the BLA (Figure 1). Control current trace is shown in Figure 1A. We demonstrate that bath application of 50 μM ALA, a lower concentration than used previously [80] (Figure 5), enhances the facilitation of GABAergic activity in the BLA in slices (Figure 1B) and this effect was reversed after wash out with control bath solution (Figure 1C). To show that the effect was not due to activation of NMDA receptors, this experiment was performed in the presence of the NMDA receptor antagonist D-AP5.

**FIGURE 2:**
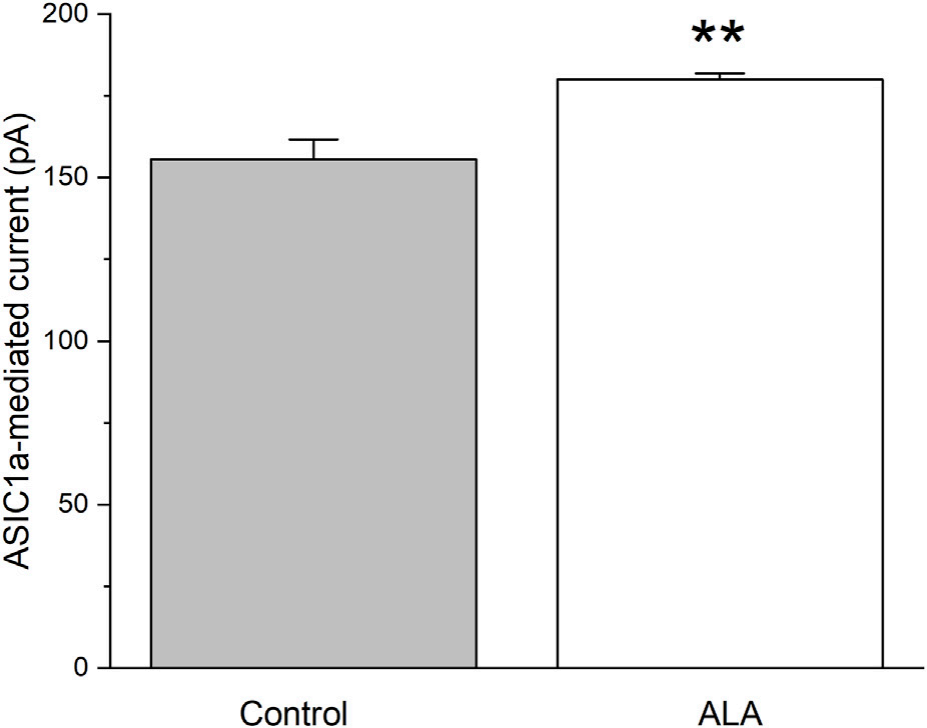
The effect of bath-applied 100 µM ALA on the amplitude of ASIC1a receptor-mediated currents evoked by pressure application of specific ASIC1a agonist NH_4_Cl. ASIC1a receptor-mediated inward currents were evoked by 40 mM NH_4_Cl applied for 300 ms at Vh = −70 mV. Ordinate axis: current amplitude in picoamperes. The increase in the amplitude of the currents was statistically significant (n = 5; t-test; p = 0.00455).

Since we have demonstrated that the ALA enhancing GABAergic inhibitory currents in the basolateral amygdala is not dependent on activation of NMDA receptors, we tested the hypothesis that this enhancing effect is dependent on the activation of ASIC1a channels. To investigate the direct effect of ALA on ASIC1a receptor (ASIC1aRs)-mediated currents at V_h_ = −70 mV, we conducted a second experiment consisting of pressure application of ammonium chloride for 300 ms, under control and bath-applied ALA (100 µM) conditions. Comparisons of ASIC1a-mediated currents, measured in picoAmpers (pA), tested under control (156 ± 6, n = 5) and 100 µM ALA (180 ± 2, n = 5) showed a significant increase (p = 0.004) when ALA was present in the bath solution (Figure 2).

**FIGURE 3:**
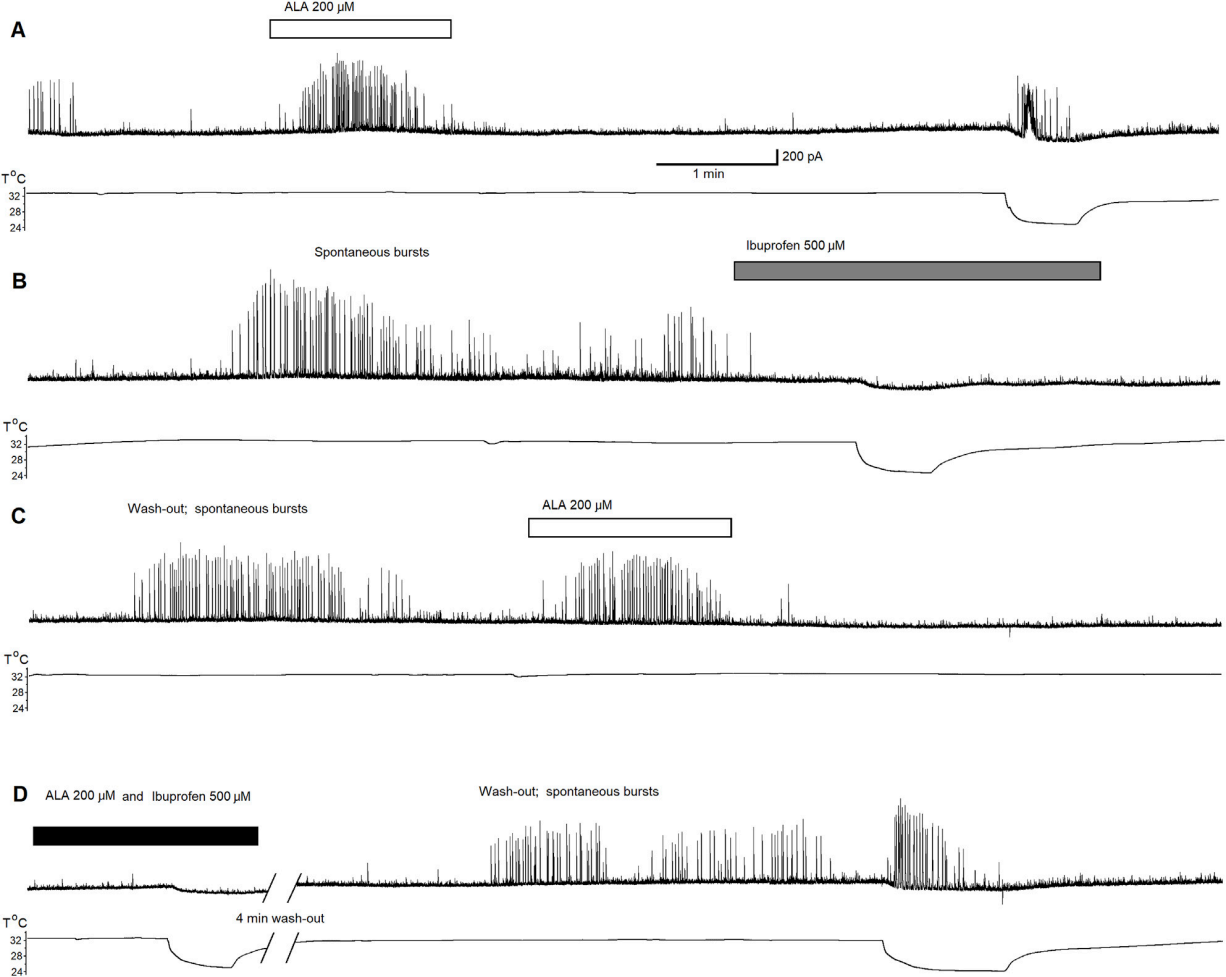
Continuous 40 min recording demonstrating initiation of ASIC1a receptor-dependent inhibitory bursts via bath-application of ALA. The recording was performed on principal BLA neurons in v-clamp mode at Vh = + 30 mV. Upper traces demonstrate currents at Vh = +30 mV in all panels. Lower traces represent changes in temperature in all panels. Bath-application of ALA (200 µM) facilitated the generation of inhibitory bursts **(A)**. Bursts were initiated also via specific facilitation of AISC1a receptors by cooling **(A)**. Spontaneous bursts in the beginning of **(B)** subsided and the cooling failed to initiate bursts in the presence of the specific ASIC1a receptor antagonist ibuprofen (500 µM). After the wash-out of ibuprofen, bath application of ALA initiated transient inhibitory bursts activity proving that the ALA effect can recover after the wash-out **(C)**. ALA application failed to induce inhibitory bursts as well as cooling also failed to initiate bursts in the presence of ibuprofen [see beginning of **(D)**]. After 4 min of wash out, spontaneous bursts reappear. Cooling initiated transient bursts activity via facilitation of ASIC1a receptors [end of panel **(D)**]. The ALA initiation of bursts most likely depended on the facilitation of ASIC1a receptors by BDNF.

To confirm that inhibitory effect of ALA is dependent on ASIC1aRs activation, we conducted a third experiment recording ASIC1a-dependent inhibitory neuronal bursts in the BLA. Figure 3 shows 40 min of continuous recording performed on pyramidal BLA neurons in v-clamp mode at V_h_ = + 30 mV. In panel Figure 3A bath-application of ALA initiates ASIC1aRs-dependent inhibitory bursts. Bursts were initiated also via specific facilitation of AISC1aRs by reducing the bath temperature, as shown in Figure 3A where the lower line is indicating drop of temperature. In Figure 3B, the bath-application of the specific ASIC1a receptor antagonist, ibuprofen (500 µM), extinguished spontaneous inhibitory bursts and when cooling was applied it failed to initiate bursts. In Figure 3C, after wash-out of ibuprofen, bath application of ALA (200 µM) initiated transient inhibitory bursts activity showing that ALA can recover bursts activity after the washout. Figure 3D shows that in the presence of ibuprofen, ALA application failed to induce inhibitory bursts as well as cooling also failed to initiate bursts. After 4 min of washout, spontaneous bursts reappear and cooling initiated transient bursts activity via facilitation of ASIC1a receptors. The pharmacological induced changes on inhibitory neuronal bursts in the BLA clearly demonstrate that the effects of ALA are dependent on ASIC1aRs activation.

**FIGURE 4:**
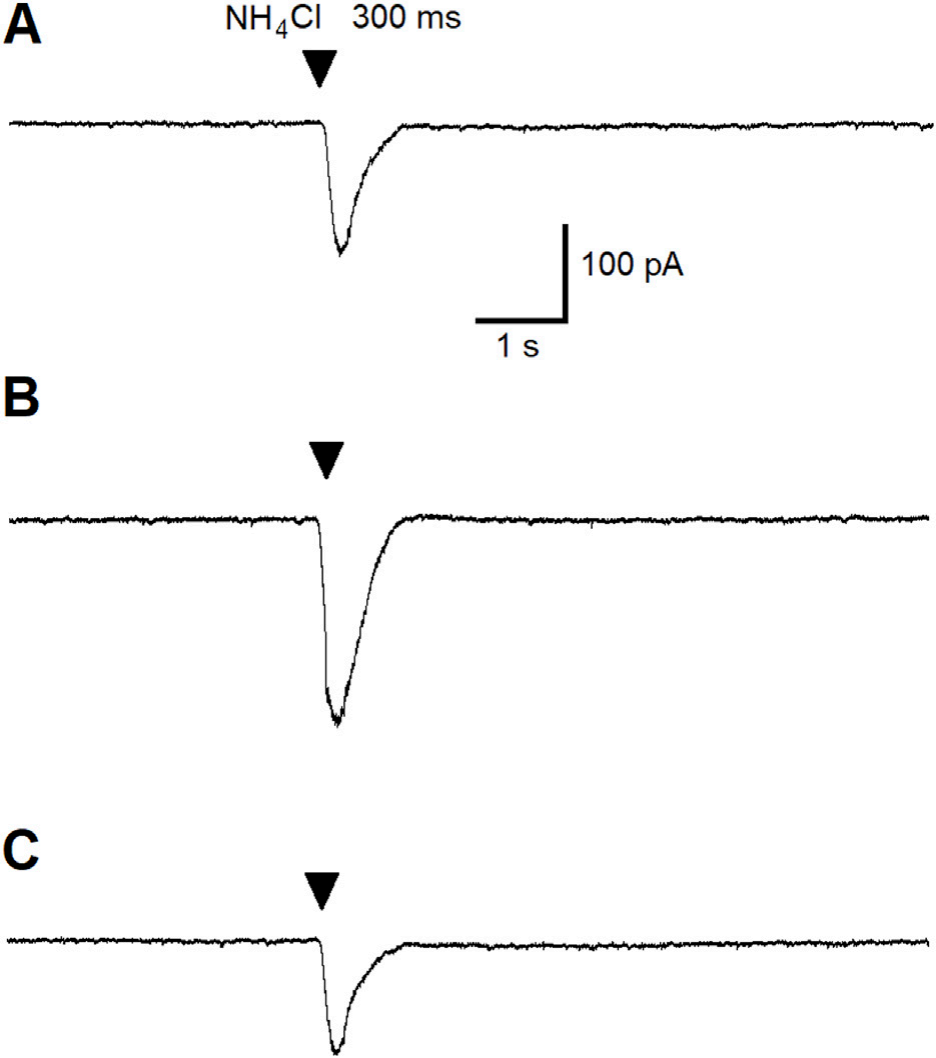
The effect of pressure-applied specific ASIC1a receptor agonist NH_4_Cl on BLA principal neurons. ASIC1a receptor-mediated currents were evoked by pressure applied NH_4_Cl (40 mM for 300 ms). Control current is demonstrated **(A)**. Bath-applied BDNF (20 ng/mL) increased ASIC1a receptor-mediated inward currents (v-clamp mode) by about 40% **(B)**. Current amplitude diminished during wash-out of BDNF **(C)**.

Since we have previously demonstrated that the ALA enhances the inhibitory GABAergic currents of pyramidal neurons through a BDNF-tyrosine receptor kinase (Trk)-mediated pathway [80] (Figure 7), we hypothesized that ALA initiation of bursts may occur due to activation of ASIC1a receptors by BDNF. Therefore, we investigated the effects of BDNF on ASIC1a currents. The specific ASIC1a receptor agonist, ammonium chloride, was pressure applied to BLA principal neurons (demonstrated by arrowheads in Figures 4A–C). Bath-applied mBDNF increased ASIC1a receptor-mediated inward currents in control (Figure 4A; v-clamp mode, V_h_ = −70 mV) by about 40% (Figure 4B) and the effect was reversed after wash-out (Figure 4C).

**FIGURE 5:**
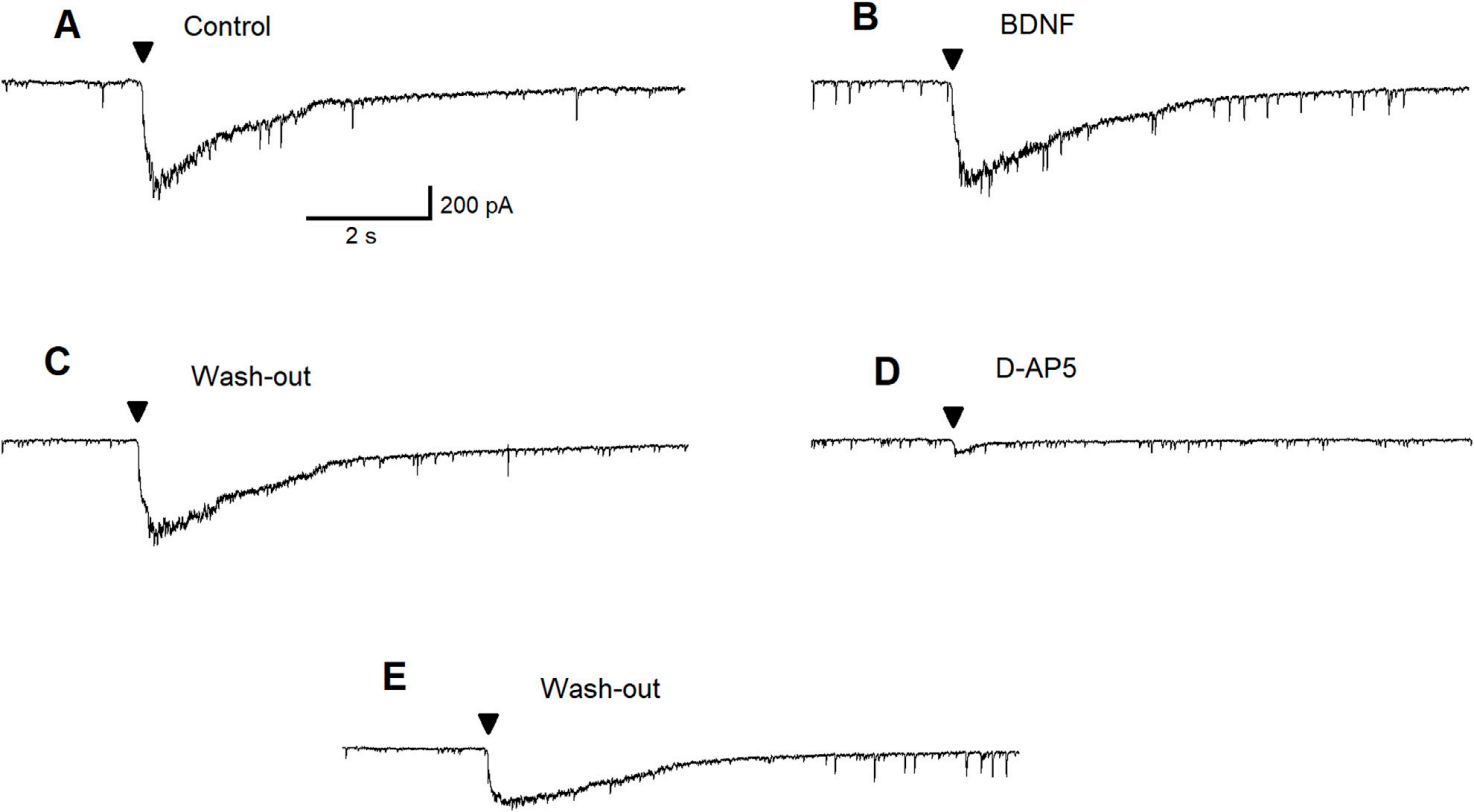
BDNF fails to facilitate NMDA-evoked currents in principal BLA neuron. Control recording of the current evoked by pressure-applied NMDA (100 µM for 100 ms, arrowheads), a subtoxic concentration **(A)**. Current evoked by pressure application of NMDA in the presence of BDNF (20 ng/mL) **(B)**. Wash out of BDNF **(C)**. Block of the current by bath applied D-AP5 (50 µM) **(D)**. Incomplete recovery of the current amplitude after wash out **(E)**.

To test the hypothesis that mBDNF may enhance NMDA receptor-mediated evoked currents in the BLA, we conducted an experiment with pressure application of NMDA in BLA principal neurons. During this experiment, the NMDA antagonist D-AP5 was not present at the control bath solution. Figure 5 shows recordings of the current evoked by pressure-applied NMDA (100 µM for 100 ms; arrowheads) under control bath-solution Figure 5A, BDNF (20 ng/mL) bath applied Figure 5B, wash-out Figure 5C, NMDA receptor antagonist D-AP5 (50 µM) bath-applied Figure 5D and wash-out Figure 5E. Results showed that NMDA receptor-mediated currents are completely inhibited by D-AP5 and there was an incomplete recovery of the current amplitude after washout. No changes in NMDA receptor-mediated currents were observed in the presence of mBDNF, demonstrating that mBDNF does not facilitate NMDA receptor-evoked currents in principal BLA neurons.

**FIGURE 6:**
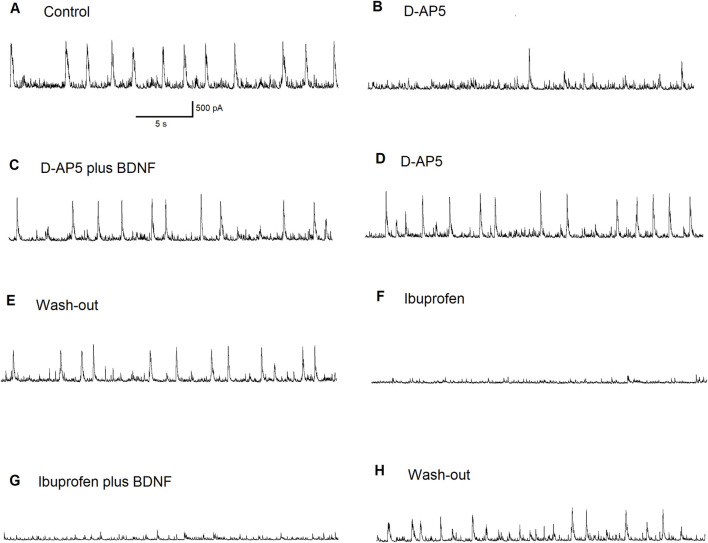
The effect of BDNF on principal BLA neuron supports the importance of ASIC1a receptors in inhibitory bursts generation. Regular inhibitory bursts have been recorded at Vh = + 30 mV. Bursts frequency was about 0.5 Hz in control **(A)**. The initial effect of the bath applied NMDA receptor antagonist D-AP5 (50 µM). NMDARs-dependent wide excitatory bursts generation was disrupted **(B)**. After 6 min in D-AP5, the addition of BDNF restored the burst generation **(C)**. Bursts persisted after wash-out of BDNF (ASIC1a receptors were involved: please note that the profile of the bursts became narrow) **(D)**. Bursts were recorded after the wash-out of D-AP5 **(E)**. The ASIC1a receptor antagonist ibuprofen (1 mM) blocked inhibitory bursts generation completely **(F)**. Addition of BDNF produced no effect **(G)**. After 10 min-long wash-out, the generation of bursts has recovered **(H)**.

Since the effects of mBDNF on inhibitory GABAergic currents in the BLA did not depend on facilitation of NMDA receptor-evoked currents, we further investigated the effects of BDNF on ASIC1a-dependent inhibitory bursts. Figure 6 shows recording of inhibitory currents in the absence of the NMDA antagonist D-AP5: Figure 6A regular excitatory bursts were recorded at holding potential (V_h_) = +30 mV and bursts frequency is about 0.5 Hz under control conditions; Figure 6B the initial effect of bath applied of the NMDA receptor antagonist D-AP5 (50 µM) shows disruption of NMDA receptor-dependent wide excitatory bursts generation; Figure 6C After 6 min in D-AP5, mBDNF (20 ng/mL) was bath-applied and results show the restoration of bursts generation; Figure 6D Bursts persisted after the washout of BDNF suggesting an ASIC1a receptor-mediated mechanism although the bursts become more narrow; Figure 6E Bursts are again recorded after the washout of D-AP5; Figure 6F the ASIC1a receptor antagonist, ibuprofen (1 mM) completely blocked inhibitory bursts generation; Figure 6G addition of BDNF shows no effect; Figure 6H After a 10 min washout, generation of bursts show recovery. The pharmacological induced changes on inhibitory neuronal bursts in the BLA principal neurons demonstrated that mBDNF restores inhibitory bursts generation that are prevented by NMDA receptor inhibition.

**FIGURE 7:**
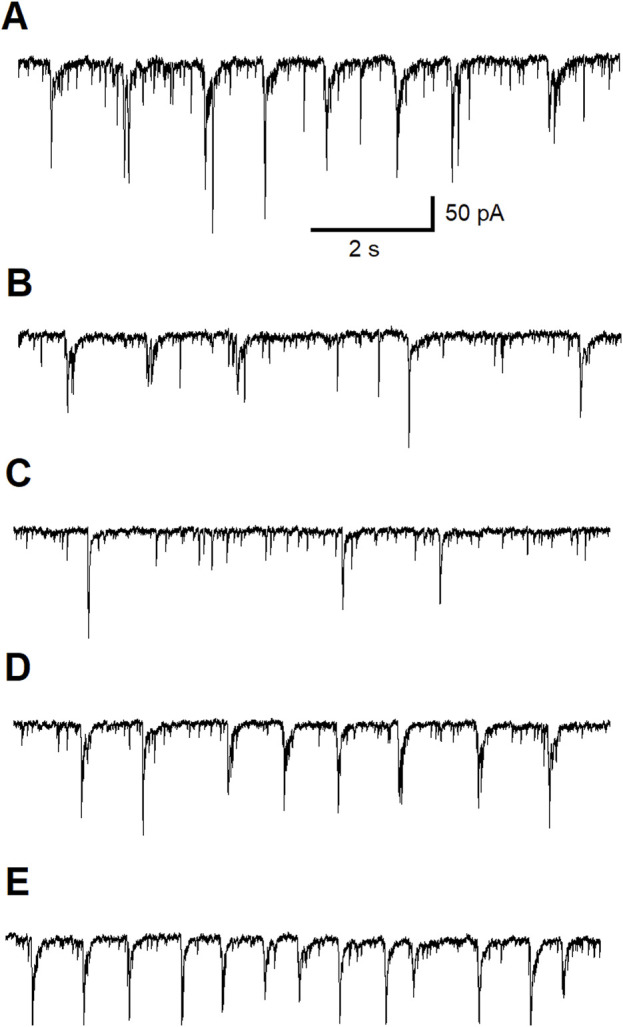
The BDNF effect on a BLA interneuron. Control: regular excitatory bursts were recorded at Vh = −70 mV; Bursts frequency was about 0.8 Hz **(A)**. The initial effect of bath applied the ASIC1a receptor antagonist ibuprofen (1 mM) **(B)**. The regularity of excitatory bursts was disrupted and the bursts frequency decreased to about 0.5 Hz. After 8 min in ibuprofen, bursts frequency decreased further to about 0.3 Hz. Bath applied BDNF (20 ng/mL) on the background of ibuprofen produced no effect **(C)**. After 10 minutes-long wash-out, regular bursts pattern was restored (bursts frequency 0.8 Hz) **(D)**. Addition of BDNF (20 ng/mL) to the bath increased bursts frequency to about 1.3 Hz **(E)**.

To confirm that mBDNF is enhancing GABAergic currents in the BLA via ASIC1aRs activation, we recorded depolarizing bursts on BLA interneurons. In Figure 7, we demonstrate that regular depolarizing bursts recorded at V_h_ = −70 mV under control bath-solution show bursts frequencies at about 0.8 Hz Figure 7A. When 1 mM of Ibuprofen, the ASIC1a receptor antagonist, was bath applied there was a reduction in bursts activity to about 0.5 Hz. After 8 min of ibuprofen application, bursts frequency decreased further to about 0.3 Hz Figure 7B. Bath application of mBDNF (20 ng/mL) on the background of ibuprofen produced no effect Figure 7C. After 10 min washout Figure 7D, regular bursts pattern was restored (bursts frequency 0.8 Hz). Addition of mBDNF to the bath Figure 7E increased bursts frequency to about 1.3 Hz. The pharmacological induced changes on inhibitory neuronal bursts in the BLA principal interneurons demonstrated that mBDNF enhances depolarizing burst activity in interneurons in the BLA, therefore increasing GABAergic inhibitory activation.

## Discussion

An epileptic focus of an idiopathic nature can develop in any area of the cortex that results in the appearance of enduring spontaneous recurrent seizures and neurocognitive and psychosocial consequences [85]. A prior brain injury, structural lesion and seizures during sleep are predisposition factors associated with seizure recurrence [2]. Seizures are transient events characterized by abnormal synchronous neuronal discharges that spread to cortical and subcortical areas in the brain [86]. Generalized epilepsy is associated with a higher rate of freedom from seizures (64–82%) compared with focal epilepsies (25–70%) during the first 2 years after diagnosis [87]. The mortality rate in epileptic individuals is 1.6–9.3 times higher than in the general population. Dire consequences of epilepsy commonly include sudden unexpected death in epilepsy, drowning, status epilepticus, and suicide [88]. Unfortunately, the seizure-free rate, defined as the absence of seizures over a 5-year period in focal and generalized epilepsy, has remained unchanged over the last twenty years despite the addition of seventeen anti-convulsants to the armamentarium of anti-seizure medications [88].

The detailed cellular and molecular mechanisms of epileptogenesis and epilepsy are unknown. Surgical removal of human epileptic tissue from patients with intractable seizures has provided insight into the electrophysiological properties of ictal and interictal events, synapse formation and characteristics of pyramidal and inhibitory interneurons. Data acquired from human epileptic tissue has shown that afferent fiber stimulation, resulting in excitatory bursts with variable latencies, were formed in the temporal or frontal cortex that depended in part on the glutamate receptor subtype, NMDA receptors [89]. Prolonged responses with after-discharges are observed in the dentate gyrus by low frequency performant path stimulation in the hippocampus from epileptic human tissue that is only found in healthy tissue when GABA_A_ receptors are partially blocked [90]. There may be decreased inhibition in dentate neurons from epileptic human tissue with hippocampal sclerosis. Single high frequency stimulation of the perforant path resulted in dentate neuronal depolarization that was amplified with the addition of a low concentration of bicuculine, a GABA_A_ receptor antagonist, suggesting that reduced inhibition may be a critical component of hyperexcitability in sclerotic hippocampal epileptic tissue [91]. Hippocampal epileptic tissue with sclerosis shows reduced GABAergic neurotransmission by fast and slow inhibitory post-synaptic potentials (IPSCs) in the dentate gyrus providing additional evidence of impaired inhibition [92].

In the absence of extracellular magnesium, a manipulation that relieves the magnesium block on the NMDA receptor-associated channel, both interictal bursts and long ictal synchronous epileptiform discharges were observed from the cortex of human epileptic brain tissue; ictal events were blocked by the NMDA receptor antagonist, 2-amino-5-phosphonopentanoic acid [APV], while non-NMDA receptor antagonists had no effect on the ictal discharges in the absence of magnesium [93]. In the hippocampus, repetitive low frequency stimulation resulted in spontaneous epileptiform discharges and were reduced in the presence of the NMDA receptor antagonist, 3-(2-Carboxypiperazin-4-yl)propyl-1-phosphonic acid (CPP). The investigators also showed spontaneous rhythmic positive polarity potentials that became more hyperpolarizing when the neuronal membrane became less negative relative to the resting membrane potential and these potentials were markedly attenuated or abolished with the addition of bicuculline. The spontaneous epileptiform discharges resulting from repetitive focal stimulation of the human epileptic tissue was associated with a reduction in the GABA_A_ receptor-mediated spontaneous rhythmic currents. These results suggest that the initiation of epileptiform discharges may be due in part to a reduced GABA_A_ receptor inhibitory-mediated mechanism even though GABA-mediated inhibition is functional in human epileptic brain tissue and confirmed in human and animal models of chronic epilepsy [94, 95]. In these cases, the human brain tissue was obtained from patients with intractable epilepsy where the tissue was described as having neuronal loss and gliosis [93]. Curiously, interictal-like discharges have been observed in the subiculum, the outflow region of the hippocampus in patients with hippocampal sclerosis. This type of activity was not detected in the CA1, CA3, dentate gyrus or entorhinal cortex. Interictal field potentials in the subiculum are significantly reduced by ionotropic glutamate and GABAA receptor antagonists [96]. Inhibitory interneurons fire just before and during interictal-like discharges. Curiously, some of the subicular pyramidal neurons have an impaired chloride homeostasis and analysis of human epileptic tissue *in vitro* confirmed the subiculum’s role in epileptogenesis [97]. It is interesting that subicular pyramidal and inhibitory interneurons abundantly express Ca_V_ 3.1 T-channels that contribute burst firing in the subiculum. That is, when T-channels are antagonized in the subiculum, burst firing changed to spike firing with low depolarizing stimuli; the absence of T-channels by genetic manipulation resulted in suppression of burst and spike firing [98].

The number of GABAergic inhibitory interneurons in the brain is relatively small (about 20%) compared with the number of excitatory neurons. Loss of GABAergic inhibitory neurons in a non-primate model of focal epilepsy was first reported by Ribak et al., (1982) [99]. More reports of GABAergic inhibitory loss or abnormalities in GABAergic function in human and animal models of status epilepticus/epilepsy followed [100–106].

When GABA_A_ receptors are activated, the chloride-associated channel opens that in turn influxes chloride. This is due to a higher extracellular concentration of chloride compared with the intracellular concentration. However, the regulation of chloride is more complex and involves the sodium-potassium-chloride (NKCCl) and potassium-chloride (KCC2) cotransporters. NKCCl increases the intracellular concentration of chloride using the sodium ion electrochemical gradient whereas KCC2 eflluxes chloride from the cell by the chemical gradient of potassium ions [107]. In normal tissue, KCC2 is highly expressed whereas the expression levels of NKCCl may be low or inhibited [108], thereby keeping the intracellular concentration of chloride low in part so that when GABA_A_ receptors are activated, the chloride-associated channel opens and chloride goes down the electrochemical gradient and influxes into the neuron. The dynamics of chloride regulation are not just a matter of an intellectual exercise. Emerging evidence suggests that inflammation is implicated in the process of epileptogenesis and drives seizure severity, frequency and excitotoxicity [109, 110]. In a recent study, a single injection of lipopolysaccharide (LPS) into the peritoneum of male and female mice, a method known to induce a cytokine cascade in the brain within 60 min after injection, leads to a significant reduction in the efflux of chloride and an uptake of chloride into neurons in the dentate gyrus and hyperexcitability and increases the probability of spike activity 24 h after injection [111]. Thus, a reversal of the normal role of the KCC2 and NKCCl cotransporters occurs that results in hyperexcitability in the brain after an episode of acute peripheral inflammation. This novel finding *in vivo* provides new mechanistic insights into epileptogenesis that may involve systemic inflammatory insults that result in an inflammatory response in brain in addition to genetics and other traditional etiologies.

An episode of status epilepticus (SE) can trigger epileptogenesis. In the kainic acid model of status epilepticus, prior work showed profound loss in GABAergic inhibitory neurons compared with the loss of excitatory neurons in the rat BLA seven to 10 days after status epilepticus. These changes were associated with an increase in the α1 GABA_A_ receptor subunit, glutamate decarboxylase (GAD), the enzyme that converts glutamate to GABA, and a decrease in the glutamate ionotropic receptor kainate type subunit 1 (GluK1). Whole-cell recordings showed a significant reduction in the amplitude and frequency of spontaneous action potential-dependent IPSCs, a reduction in the frequency but not amplitude of miniature IPSCs and an impairment in the modulation of IPSCs via GluK1-containing receptors [112]. These results underscore the striking vulnerability of GABAergic inhibitory interneurons after SE that is not compensated by surviving GABAergic inhibitory neurons that expressed increased levels of the α1 subunit of the GABA_A_ receptor and the increase in GAD. These alterations may set the stage of the development of an epileptic focus due to the loss of GABAergic inhibitory neurons in the rat BLA.

Because loss or impairment of GABAergic function has been implicated in human and animal models of temporal lobe epilepsy, compounds that positively affect the GABAergic system would be beneficial in controlling and/or preventing epilepsy. Anticonvulsants that enhance GABAergic function have been approved by the FDA such as valproic acid, and lamotrigine and are already in use today. Unfortunately, many of the anticonvulsants have side effects, with some that are serious and/or debilitating [88].

Alpha-linolenic acid is an omega-3 essential polyunsaturated fatty acid (PUFA) found in plants including flaxseed, nuts, and vegetable oils [113]. In contrast to anticonvulsant drugs, ALA has a wide safety margin There is ample literature to suggest that omega-3 polyunsaturated fatty acids, including alpha-linolenic acid, have therapeutic potential for neurologic and psychiatric disorders. Therapeutic efficacy of ALA has been observed in animal models of stroke [114–119] that improves outcome [120], spinal cord injury [121], kainic acid-induced status epilepticus [81], a temporal lobe epilepsy model, after soman-induced status epilepticus [122] that reduces behavioral and cognitive impairment [123, 124] in part via an mammalian target of rapamycin-mediated mechanism [125] and in a mild traumatic brain injury model [126]. ALA is metabolized to oxylipins by exposure to air [84], lipoxygenase, and cyclooxygenase pathways [127]. Polyunsaturated fatty acids also undergo metabolism by the CYP450 pathway [128]. A new report showed that ALA is metabolized to linotrins and these oxylipins exert anti-inflammatory properties in cultured microglia exposed to lipopolysaccharide [113].

Administration of three doses of ALA at 30 min, 3 days and 7 days after injury was originally found to enhance brain plasticity including a two-fold increase in mBDNF in the hippocampus and cortex, two brain regions involved in neuronal plasticity, a significant increase in neurogenesis in the subgranular zone of the dentate gyrus, an increase in expression in key proteins involved in synaptogenesis and glutamate neurotransmission; This same dosing schedule also exerts an anti-depressant effect [129]. The administration of three doses or subchronic treatment of ALA was used to show neuroprotective efficacy in animal models of stroke, mTBI and soman-induced status epilepticus. It’s been known for about 20 years that ALA activates a neuronal TREK (TWIK-related potassium channel)-1 channel. TREK-1 channels are 2-pore domain background potassium channels that are open at membrane potentials and likely contribute to the resting membrane potential [130]. Activation of TREK-1 channels by ALA hyperpolarizes the membrane to advantage the magnesium block and reduce NMDA receptor activation on post-synaptic membranes as well as reduce the excessive release of glutamate from presynaptic sites. Activated TREK-1 channels by ALA are also involved in cerebral vasodilation to increase blood flow and protect against stroke [117].

There are some reports showing that ALA reduces seizures. An early study showed that a mixture of ALA and linoleic acid, an omega-6 PUFA, in a ratio of 1:4 administered over 3 weeks prior to exposure to four different models of seizures reduced the seizure latency 22-fold in up to 84% in the number of rats with seizures and up to a 97% reduction in the duration of seizures [131]. Recently, intra-gastric administration of ALA for 40 days after injection of pentylenetetrazol (PTZ), a convulsant compound, reduced the frequency of epileptic seizures, improved the cognitive and behavior impairment and reduced neuronal apoptosis via downregulating the JAK/STAT-3 pathway [132]. The exact mechanisms of how ALA reduces the various elements of seizures i.e., duration, frequency, latency, are unknown.

We have shown previously that a single dose of ALA (1500 nmol/kg) injected subcutaneously into male Sprague-Dawley rats increased the charge transfer of inhibitory postsynaptic potential currents mediated by GABA_A_ receptors in pyramidal neurons by 52% in the BLA and by 92% in the CA1. Bath application of ALA also increases the facilitation of GABA_A_ receptor-mediated neurotransmission in the BLA and CA1 subfield of the hippocampus in naïve male rats. Interestingly, K252a, the high affinity and selective TrkB inhibitor, completely blocked the ALA-induced increase in GABAergic neurotransmission in the BLA and CA1, suggesting an mBDNF-mediated mechanism. Bath application of mBDNF (20 ng/mL) also enhanced GABAergic inhibitory activity in the BLA and CA1 pyramidal neurons similar to what was observed with ALA [80]. We proposed that low-level activation of NMDA receptors results in the immediate release of mBDNF from either presynaptic neurons [133] or astroglial cells [134] to mediate this effect.

Here, we show that bath application of ALA elicits ASIC1a-dependent GABA_A_ receptor-mediated inhibitory bursts located on glutamatergic and GABAergic inhibitory neurons in rat BLA slices. Because we suggested that the enhanced facilitation of GABAergic neurotransmission was elicited via a TrkB-mediated mechanism, we tested whether mBDNF might be involved in the ASIC1a-dependent GABAergic inhibitory bursts. To this end, mBDNF (20 ng/mL) was bath applied in the presence of the NMDA receptor antagonist, D-AP5. Because the effects of mBDNF on inhibitory GABAergic currents in the BLA did not depend on activation of NMDA receptor-evoked currents, we investigated the effects of mBDNF on ASIC1a-dependent inhibitory bursts. Activation of NMDA receptor-evoked currents elicit inhibitory bursts. However, in the presence of the NMDA receptor antagonist, D-AP5, NMDA receptor-mediated excitatory bursts showed disruption. Mature BDNF (20 ng/mL) was bath applied 6 minutes after the addition of D-AP5 and results showed restoration of bursts generation (Figure 6). The bursts persisted after the washout of mBDNF suggesting an ASIC1a receptor-mediated mechanism and this mechanism was confirmed when the ASIC1a receptor antagonist, ibruprofen (1 mM) was added and inhibitory bursts generation was completely blocked. Washout resulted in the return of bursts. These results show that mBDNF restores inhibitory burst generation in the presence of AP-5, an NMDA receptor antagonist.

To provide confirmatory data that mBDNF enhances GABAergic currents in the BLA via ASIC1aR activation, depolarizing bursts were recorded on BLA interneurons. Burst frequencies in the bath solution recorded at a holding voltage of −70 mV show bursts frequencies about 0.8 Hz; addition of Ibuprofen reduced bursts activity to about 0.5 Hz. Eight minutes later, bursts frequency decreased further to about 0.3 Hz. Addition of mBDNF to the bath on the background of ibuprofen showed no effect. Washout for 10 min showed regular bursts pattern restoration (bursts frequency 0.8 Hz) and bath application of mBDNF increased bursts frequency to about 1.3 Hz. These results show that mBDNF enhances depolarizing burst activity in interneurons in the BLA, thereby enhancing GABAergic inhibitory activation.

The amygdala is critically involved in TLE and shows extensive damage in TLE patients. The amygdala’s reciprocal connections with the hippocampus and other temporal structures likely mediate the spread of the seizure focus. In addition, the amygdala may be the location of the seizure focus as kindling produces seizures in the amygdala faster than the hippocampus and the earliest interictal spikes or epileptiform discharges occur in the amygdala even if kindling was performed in the hippocampus. Moreover, BLA activation is especially implicated in the generation of status epilepticus in animal models of seizures even when the seizures are generated in extra-amygdalar brain regions. Our results show that ALA enhances the facilitation of GABAergic inhibitory activity and initiate GABAergic inhibitory bursts via the facilitation of ASIC1a channels. Because ALA increases GABAergic inhibitory bursts directly on inhibitory neurons and indirectly via activation of ASIC1a channels located on glutamatergic neurons in the BLA and activation of ASIC1a channels produce an overall reduction in neuronal excitability, we suggest that chronic administration of ALA, an omega-3 PUFA with a wide safety margin may reduce seizure activity in the BLA via enhancing GABAergic inhibitory activity and by facilitating the activation of ASIC1a channels. We showed previously that ALA enhances GABAergic inhibitory activity in the CA1 subfield in the hippocampus. Our results may also explain recent results showing that ALA exhibits anti-convulsant properties in generalized seizures [132].

## Data Availability

The raw data supporting the conclusions of this article will be made available by the authors, without undue reservation.
